# Buffering growth variations against water deficits through timely carbon usage

**DOI:** 10.3389/fpls.2013.00483

**Published:** 2013-11-28

**Authors:** Florent Pantin, Anne-Laure Fanciullino, Catherine Massonnet, Myriam Dauzat, Thierry Simonneau, Bertrand Muller

**Affiliations:** ^1^UMR 759, Laboratoire d’Ecophysiologie des Plantes sous Stress Environnementaux, Institut de Biologie Intégrative des Plantes, Institut National de la Recherche AgronomiqueMontpellier, France; ^2^UR 1103, Génétique et Ecophysiologie de la Qualité des Agrumes, Institut National de la Recherche AgronomiqueSan Giuliano, France; ^3^UR 1115, Plantes et Systèmes de Culture Horticoles, Institut National de la Recherche AgronomiqueAvignon, France

**Keywords:** leaf growth, fruit growth, water deficit, carbon starvation, carbon satiation, starch metabolism, VPD

## Abstract

Water stresses reduce plant growth but there is no consensus on whether carbon metabolism has any role in this reduction. Sugar starvation resulting from stomatal closure is often proposed as a cause of growth impairment under long-term or severe water deficits. However, growth decreases faster than photosynthesis in response to drought, leading to increased carbohydrate stores under short-term or moderate water deficits. Here, we addressed the question of the role of carbon availability on growth under moderate water deficits using two different systems. Firstly, we monitored the day/night pattern of leaf growth in *Arabidopsis* plants. We show that a moderate soil water deficit promotes leaf growth at night in mutants severely disrupted in their nighttime carbohydrate availability. This suggests that soil water deficit promotes carbon satiation. Secondly, we monitored the sub-hourly growth variations of clementine fruits in response to daily, natural fluctuations in air water deficit, and at contrasting source–sink balances obtained by defoliation. We show that high carbohydrate levels prevent excessive, hydraulic shrinkage of the fruit during days with high evaporative demand, most probably through osmotic adjustment. Together, our results contribute to the view that growing organs under moderate soil or air water deficit are not carbon starved, but use soluble carbohydrate in excess to partly release a hydromechanical limitation of growth.

## INTRODUCTION

Water stress critically impairs plant growth and affects primary productivity worldwide (Boyer, 1982; Zhao and Running, 2010). Understanding the mechanisms by which growth decreases under water stress is a long-standing matter of debate (Hsiao, 1973; Tardieu et al., 2011). Growth can be both defined as an irreversible increase in volume – expansive growth – and an accumulation of biomass into new structures – structural growth (reviewed in Pantin et al., 2012). During expansive growth, turgor pressure exceeds the resistance offered by the cell walls, leading to an enlargement of the walls and a net influx of water into the growing cells (Lockhart, 1965; Cosgrove, 1993). Structural growth is tightly dependent upon carbon supply to growing tissues, which are sites of intensive respiration (Bidel et al., 2000) and biosynthesis of carbon compounds essential for cell growth, such as cellulose, hemi-cellulose, or proteins (Smith and Stitt, 2007; Gibon et al., 2009). Under water stress, both water relations and carbon balance are impaired, because plants close their stomata to limit transpirational water loss, which also limits the carbon entry required for photosynthesis. Accordingly, plant growth under water stress could be reduced either because its water relations are unsuitable for growth – hydromechanical limitation – or because its carbon balance is low – metabolic limitation.

It has been often proposed that growth under water stress was modulated by hydromechanical constraints. Under water scarcity, water flux to growing cells is reduced because water potential gradients are disrupted (Tang and Boyer, 2002). Aquaporin closure by drought signals may also worsen the delivery of water to the growing tissues (Parent et al., 2009; Shatil-Cohen et al., 2011; Pantin et al., 2013). This leads to a drop in turgor pressure, that plants may counteract through osmotic adjustment, which partly relies on recruiting carbon solutes in the vacuole. Finally, water deficits tend to stiffen the cell walls (Fan et al., 2006; Zhang et al., 2011), making turgor pressure less efficient in driving growth. Thus, the hydromechanical limitation of growth by water deficits arises from an imbalance between the force required to enlarge the cell walls and the turgor pressure, which can be modulated by the mobilization of organic solutes.

The growth rate of sink organs is also tightly coordinated with carbon availability and this holds for roots (Freixes et al., 2002; Yazdanbakhsh and Fisahn, 2010; Willaume and Pagès, 2011; Yazdanbakhsh et al., 2011), fruits (Génard et al., 1998; Lescourret et al., 1998; Léchaudel et al., 2005; Andriotis et al., 2012), flowers (Smith and Stitt, 2007; Dosio et al., 2011), or young leaves (Muller et al., 2001; Pantin et al., 2011). Moreover, under moderate water deficits, carbohydrate concentrations increase in growing tissues due to an uncoupling between growth rate and photosynthesis, the former being more sensitive than the latter (Muller et al., 2011). These differences of sensitivities have been reported for a long time (Boyer, 1970), and observed in a variety of species (Sadras and Milroy, 1996), including trees (Bogeat-Triboulot et al., 2007). They may at least partly originate from the strong resilience of the metabolic component of photosynthesis to water deficit (Kaiser, 1987; Flexas et al., 2004), while expansive growth is probably the most sensitive process to water stress (Hsiao, 1973). As a result, the correlations between growth rate and carbohydrate concentrations disappear under moderate water deficits (Muller et al., 2011).

These observations hold for moderate water deficits. Contrastingly, under long-term or severe water deficit, photosynthesis can be so severely reduced that plants may enter into carbon starvation, therefore provoking a metabolic limitation of growth or even plant death (McDowell, 2011; Quirk et al., 2013; Sevanto et al., 2013). Carbon starvation could be particularly critical for isohydric species which prevent excessive drop of their leaf water potential by an early closure of their stomata (McDowell et al., 2008).

Increases in carbohydrate concentration following moderate water stress raise the question of the role of carbohydrate availability on plant growth under water deficit. In this paper, we addressed this question by analyzing how organs under moderate water stress partition their growth at a sub-daily time-scale. Plants experience a diurnal variation of carbon and water availability: during the night, metabolism relies on transitory pools of carbon such as starch, which set a metabolic limit to nighttime growth; these transitory pools are synthesized within the leaves in the daytime, during which transpiration competes with growth for water (Pantin et al., 2012). We thus took advantage of this daily fluctuations using two different systems and contrasting regimes of water and carbon availability. Firstly, we analyzed leaf growth at a day/night time-step in *Arabidopsis* mutants impaired in carbohydrate metabolism and grown under soil water deficit. Secondly, we studied the sub-hourly growth pattern of clementine fruits grown under various source–sink regimes and exposed to daily, natural fluctuations of air water deficit. Our results support the view that water deficits promote carbon satiation of sink organs and that the main effect of carbon availability on organ growth under moderate water deficits is through the supply of organic osmotica for turgor maintenance.

## MATERIALS AND METHODS

### DAY/NIGHT PATTERNS OF LEAF GROWTH IN *Arabidopsis* USING THE PHENOPSIS PLATFORM

*Arabidopsis thaliana* plants were grown in soil at a 16-h photoperiod (PAR = 170 μmol m^-^^2^ s^-^^1^) using the phenotyping platform PHENOPSIS, that allowed both precise control of the water content of each pot and imaging of the plant from the top (Granier et al., 2006). The soil water content was maintained at a well-watered level of 0.40 g_water_ g^-1^_dry soil_ (equivalent to a predawn water potential of -0.2 MPa), the vapor pressure deficit (VPD) at 0.8 kPa, and the temperature at 20°C. When plants reached the phenological stage 1.02 (Boyes et al., 2001), for half of the plants, irrigation was suspended until soil water content reached a target value corresponding to a moderate water stress (0.23 g_water_ g^-1^_dry soil_, equivalent to a predawn water potential of -0.7 MPa). Photographs taken at the end of each day and night, combined to a simplified version of the image analysis procedure described in Pantin et al. (2011), allowed us to monitor the day/night relative elongation rate of the sixth leaf during 8 days following leaf emergence, corresponding to the time at which this leaf reaches the half of its final size in the wild-type Col-0 under control conditions.

**FIGURE 1:**
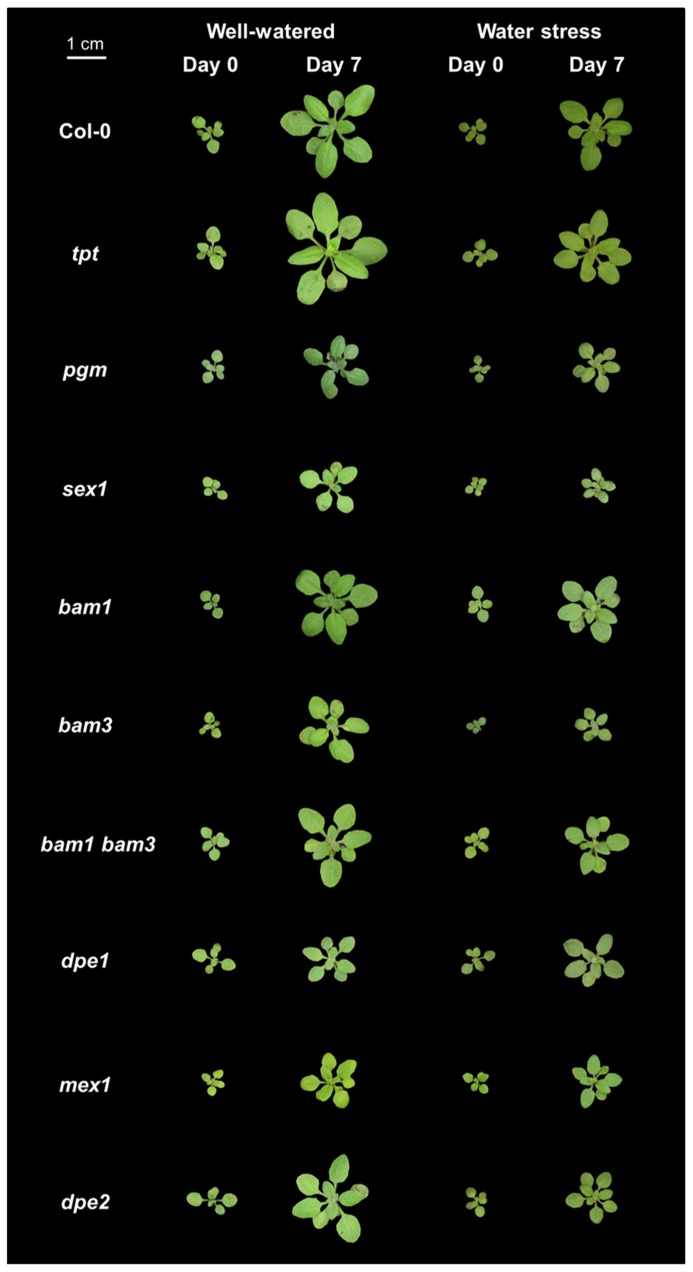
**Photographs of representative plants of *Arabidopsis* mutants affected in carbon metabolism grown in well-watered conditions or under soil water stress.** The wild-type Col-0 is shown at day 0 and day 7 following emergence of the sixth leaf in well-watered conditions or under moderate water stress (predawn water potential of -0.7 MPa), together with a mutant affected in the daytime translocation of chloroplastic photosynthates (*tpt*), starch metabolism (*pgm*), or starch utilization at night (*sex1*, *bam1*, *bam3*, *bam1 bam3*, *dpe1*, *mex1*, *dpe2*).

Both the wild-type accession Col-0 and mutants affected in carbon metabolism were studied. These mutants were affected either in the daytime translocation of chloroplastic photosynthates (*tpt*), in starch synthesis (*pgm*), or in starch utilization at night (*sex1*, *bam1*, *bam3*, *bam1 bam3*, *dpe1*, *mex1*, *dpe2*). All mutants were in the Col-0 background. The genotypes are shown in **Figure 1** at day 0 and day 7 following the emergence of the sixth leaf, in both well-watered and water stress conditions.

### DAILY PATTERNS OF FRUIT GROWTH IN CLEMENTINE USING DISPLACEMENT TRANSDUCERS IN THE FIELD

We analyzed the growth pattern of clementine fruits in an experimental orchard during the 2008–2009 season. We used 18-year-old clementine trees (*Citrus clementina* Hort. ex Tan.) which were all clonal replicates grafted on Carrizo-citrange (*Citrus sinensis* [L.] Osbeck × *Poncirus trifoliata* Raf.) and grown near San Giuliano in Corsica (42° 18′ 55″ N, 9° 29′ 29″ E; 51 m a.s.l.). Trees were about 2.5 m high, and were spaced at 4 m × 6 m. The plants were subjected to standard cultural practices for commercial clementine production. Fertilizers were supplied and insects and diseases controlled according to the recommendations of the local agriculture department. The trees were irrigated at full water requirements using micro-sprinklers under the canopy. Irrigation was scheduled based on evapotranspiration calculations estimated from the Penman–Monteith equation (Monteith, 1965) and from information supplied by the local weather station. Microclimate around the fruits was monitored using thermocouples, sensors measuring global solar irradiance (CES180, Cimel Electronique, Paris, France) and relative humidity sensors (HMP45C, Campbell, Scientific Inc., UT, USA). Climate data were stored every 15 min. Fruit growth was monitored using displacement transducers recording variations in fruit diameter during 45 days between early September and late November 2008. Climate sensors and displacement transducers were connected to the same control box and data-logger (21X Micrologger, Campbell Scientific Inc., UT, USA), allowing simultaneous measurements for growth and climate every 15 min.

To modify the carbon availability for the growing fruits, a defoliation treatment was applied on selected fruiting branches in order to modify the leaf-to-fruit ratio. During the flowering period, the plants were isolated from pollinators to prevent cross-pollination and seed production. Only fully expanded flowers from May 5th to May 15th 2008 were selected to obtain fruits of similar age. After the completion of cell division in the fruit (at the end of July under Mediterranean climate; Tadeo et al., 2008), the leaf-to-fruit ratio was set to 30, 15, or 5 leaves per fruit to obtain a control, moderate or low carbon availability (Poiroux-Gonord et al., 2013). The 120 selected fruiting branches were composed of 1-year shoots from the spring flush of the previous season. Fruiting branches were chosen among the trees as having similar initial stem diameter (about 1 cm), height above ground (about 1.5 m), and exposure to light in East orientation. Before the defoliation treatment, girdling was applied on shoots with at least 30 leaves in July, after fruit set. Girdling consisted of removing a 10 mm-wide band of bark in the middle of the main stem of each selected branch to prevent any movement of assimilates between the fruiting branch and the rest of the tree. Leaves were all fully expanded at the time of girdling. Fruit growth was monitored on at least three fruits per level of leaf-to-fruit ratio. Displacement transducers were also placed on three peeled fruits from girdled fruiting branches with 30 leaves.

The fresh and dry weight of the pulp and the peel were recorded regularly from September to March, together with glucose, fructose, and sucrose contents of the pulp. Immediately after harvest, fruits peel and pulp were weighed separately for fresh mass determination. Aliquots were lyophilized for dry mass measurements and sugar analyzes. Then, the pulp material was ground to a fine powder and stored at -80°C for subsequent analyzes. Sugar analyzes of the pulp were performed by HPLC according to the method previously described in Poiroux-Gonord et al. (2013).

### STATISTICAL ANALYZES

All graphics and statistical analyzes were performed with the R software (R Development Core Team, 2012).

To analyze the day/night pattern of relative elongation rate of the sixth leaf in the ten *Arabidopsis* genotypes under well-watered conditions and under water stress, a heat map was performed as follows. For each genotype × environment combination, the difference between nighttime- and daytime-mean relative elongation rate was computed at each of the 8 days after leaf emergence, and assigned to a color between green (daytime growth) and red (nighttime growth). A hierarchical clustering of the kinetics was then performed using the Euclidean distances. The original daytime and nighttime values were further detailed for the days 4–6 following leaf emergence, as a bar plot showing the means and the 95% confidence intervals.

To analyze the effect of the natural variations in VPD on clementine fruit growth, we took advantage of the natural variability in the daily patterns of VPD collected throughout the experiment, which were highly variable from day to day. Each day starting at 00:00 and ending at 23:59 was considered as an individual with 96 variables, corresponding to the VPD collected every 15 min. A k-means clustering analysis was performed on these climate data, which allowed to statistically allocate the days according to their similarity in VPD. Three clusters were obtained, equivalent to a day with high (the daily maximal value of VPD, VPD_max_ > 1 kPa), moderate (0.5 kPa < VPD_max_ < 1 kPa), and low VPD (VPD_max_ < 0.5 kPa), respectively. The fruit growth patterns (as well as PAR measurements) were then affected to one of these three clusters according to the day they were collected, and were averaged within each cluster on a 15 min basis.

## RESULTS AND DISCUSSION

### GROWTH LIMITATION UNDER SOIL WATER DEFICIT: INSIGHTS FROM *Arabidopsis* LEAVES

#### Growth of young leaves is driven by carbon availability in well-watered conditions

**FIGURE 2:**
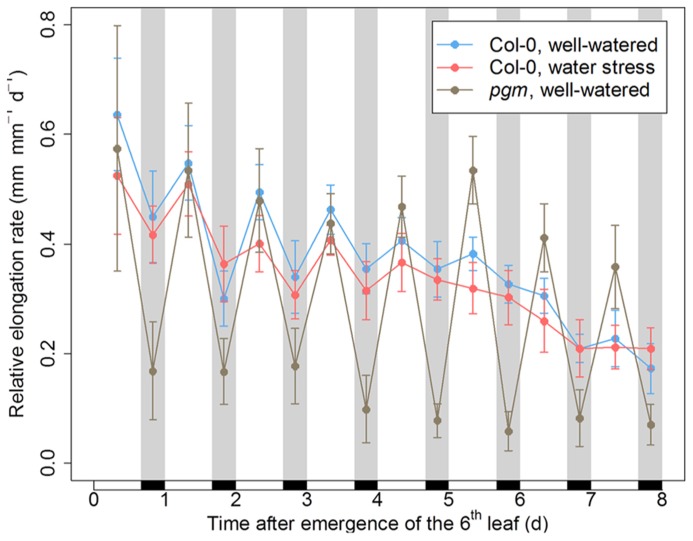
**Day/night pattern of leaf growth in *Arabidopsis* plants with a disturbed hydraulic or metabolic status.** The relative elongation rate of the sixth leaf was monitored for 8 days following its emergence in the wild-type Col-0 under well-watered conditions or under moderate soil water stress (predawn water potential of –0.7 MPa), as well as in the starchless mutant *pgm* under well-watered conditions. Black rectangles and gray bands indicate the night periods. Error bars are 95% confidence intervals.

To analyze the impact of the day/night regime on leaf growth, we monitored the elongation rate of the sixth leaf in *Arabidopsis* plants during the first 8 days and nights following its emergence. In the wild-type Col-0, nocturnal depressions of growth were observed early after leaf emergence, but progressively vanished during leaf development (**Figure 2**). In *Arabidopsis* leaves, metabolic demand at night is sustained by the transitory starch synthesized in the daytime, a period during which stomatal transpiration reduces water availability. Accordingly, using developmental patterns of leaf relative expansion rate collected on several *Arabidopsis* genotypes grown under different environmental conditions, we showed previously that nighttime depressions of growth are associated to a metabolic limitation, while daytime depressions of growth are related to a hydraulic limitation (Pantin et al., 2011). Thus, the result shown in **Figure 2** are consistent with the idea that a metabolic limitation exerting on leaf growth at night is progressively released as the leaf switches from sink to source (Pantin et al., 2011).

**FIGURE 3:**
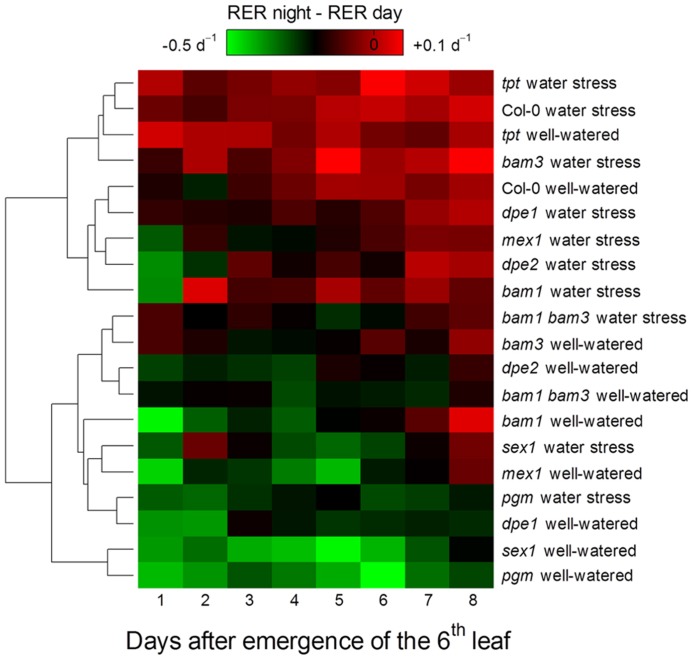
**Heat map of the day/night patterns of leaf growth in *Arabidopsis* mutants affected in carbon metabolism and grown in well-watered conditions or under moderate soil water stress.** The dendrogram represents a hierarchical clustering analysis (Euclidean distances) of the difference between nighttime and daytime relative elongation rate of the sixth leaf during 8 days following its emergence. The difference was associated to a color, with closeness to green indicating growth preferentially in the daytime and closeness to red indicating growth preferentially in the nighttime. Note that moderate soil water stress increases nighttime growth relative to daytime growth in all genotypes.

**FIGURE 4:**
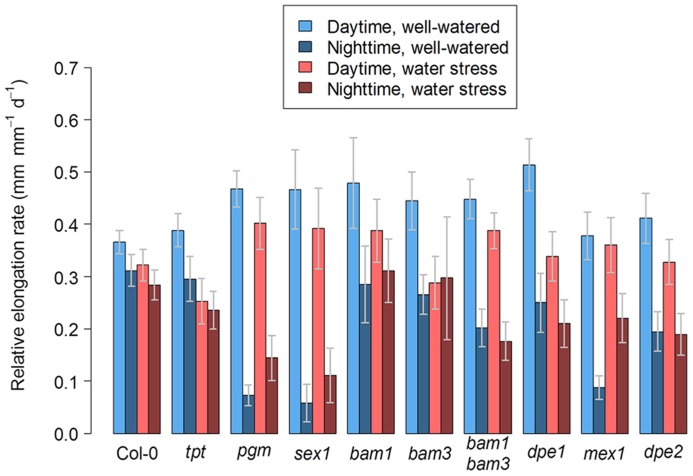
**Day/night leaf growth in *Arabidopsis* mutants affected in carbon metabolism and grown in well-watered conditions or under soil water stress.** The bar plot shows the daytime and nighttime growth averaged for the days 4, 5, and 6 following emergence of the sixth leaf under well-watered conditions or under moderate soil water stress. Error bars are 95% confidence intervals.

To study how a perturbation in carbon availability may affect leaf growth, we analyzed the leaf growth pattern in several mutants affected in starch metabolism or in photosynthate translocation. Photographs of these mutants are shown in **Figure 1**. For all genotypes, the difference between nighttime and daytime relative elongation rate during leaf development is presented in **Figure 3** as a heat map. The genotypes were then ranked according to a clustering analysis performed on these day/night variations of elongation. The daytime and nighttime leaf elongation observed during the days 4, 5, and 6 following emergence of the sixth leaf were also averaged and presented in **Figure 4**.

In well-watered conditions, the night reduction of elongation of the* tpt* mutant in the early stages was less than in the wild-type (**Figure 3**), as already observed in potato (Kehr et al., 1998). This is consistent with the mutant’s impairment in the daytime translocation of chloroplastic photosynthates, which affects daytime carbon availability but increases starch synthesis (Schneider et al., 2002; Cho et al., 2011). Conversely, all mutants affected in nighttime carbon availability (i.e., through an impaired starch synthesis or breakdown) showed marked depressions of growth in the nighttime, as illustrated with* pgm* in **Figure 2** and with all genotypes in **Figures 3** and **4**. Compared to the wild-type, these nocturnal depressions were both amplified in magnitude and extended to later stages of leaf development. The most severe phenotype was observed in* pgm* and* sex1*, namely the genotypes of our list which were affected the most upstream in the starch metabolism pathway, and which were the most dramatically impaired in starch turnover (Zeeman et al., 2010; Stitt and Zeeman, 2012). The other starch mutants, *dpe1*, *dpe2*, *mex1*, * bam1*, * bam3*, and the* bam1 bam3* double mutant, clustered closely together and showed a less extreme phenotype than *pgm* or *sex1* (**Figure 3**). Overall, this ranking of mutants was in agreement with their intermediate impairment in starch degradation (Critchley et al., 2001; Messerli et al., 2007; Fulton et al., 2008), though *bam1* was not expected to have a phenotype distinct from the wild-type since this mutant has a normal day/night pattern of starch turnover and that BAM1 is presumably active in guard cells during the daytime (Fulton et al., 2008; Valerio et al., 2011). It may be argued that this classification could be biased by an extended duration of leaf growth in these mutants. However, a similar classification of starch mutants was obtained in Pantin et al. (2011), where the growth patterns were normalized according to the duration of development. Thus, in well-watered conditions, the day/night pattern of leaf growth was globally dictated by the severity of the impairment in daytime or nighttime carbohydrate availability.

#### Soil water deficit releases the metabolic limitation on leaf growth

When Col-0 plants were grown under moderate soil water stress, shoot area decreased (**Figure 1**). Elongation of the sixth leaf decreased especially in the daytime (**Figure 2**), although this effect was less significant than in Pantin et al. (2011) due to the higher evaporative demand prevailing in the growth chamber used in the latter study. Strikingly, under water stress, all mutants affected in nighttime carbon availability increased nighttime growth relative to daytime growth (**Figure 3**). This was achieved by maintaining (*dpe1*, *dpe2*, *bam1*, * bam3*, *bam1 bam3*) or even increasing (*pgm*, *sex1*, *mex1*) nighttime growth rates while decreasing daytime growth rates (**Figure 4**). As a consequence, water-stressed *pgm* and *sex1 *clustered with the well-watered genotypes less affected in nighttime carbon availability, while the latter genotypes under water stress tended to cluster closer to the wild-type (**Figure 3**). This result indicates that water stress partly releases the carbon limitation on leaf growth that these mutations generate under well-watered conditions, either by providing structural growth with molecular bricks or by fuelling expansive growth with osmotica.

This result also shows that the accumulation of carbohydrates observed under moderate water stress (e.g., Hummel et al., 2010) in the wild-type does not translate into additional growth as it does in the starch mutants at night. It could be argued that carbohydrates are not available for structural growth if sequestered in the vacuole for osmotic purposes, thereby generating a metabolic limitation under water stress in the wild-type. However, in the model plant *Arabidopsis* under moderate water deficit, osmotic adjustment mobilizes only a minor part of the daily carbohydrate balance, which was largely in excess due to reduced growth but maintained photosynthesis (Hummel et al., 2010). Thus, our results support the conclusion that the reduction of leaf growth observed in wild-type plants under moderate water stress does not arise from carbon starvation; by contrast, moderate water deficits induce carbon satiation.

### GROWTH LIMITATION UNDER ATMOSPHERIC WATER DEFICIT: INSIGHTS FROM CLEMENTINE FRUITS

#### Carbon availability positively affects fruit growth and sugar contents

To further investigate the role of carbon availability on growth of organs when exposed to moderate water deficit, we analyzed the growth pattern of clementine fruits with contrasting leaf-to-fruit ratio (30, 15, or 5) in an experimental orchard, namely in field conditions. Trees were all well-watered and fertilized.

**FIGURE 5:**
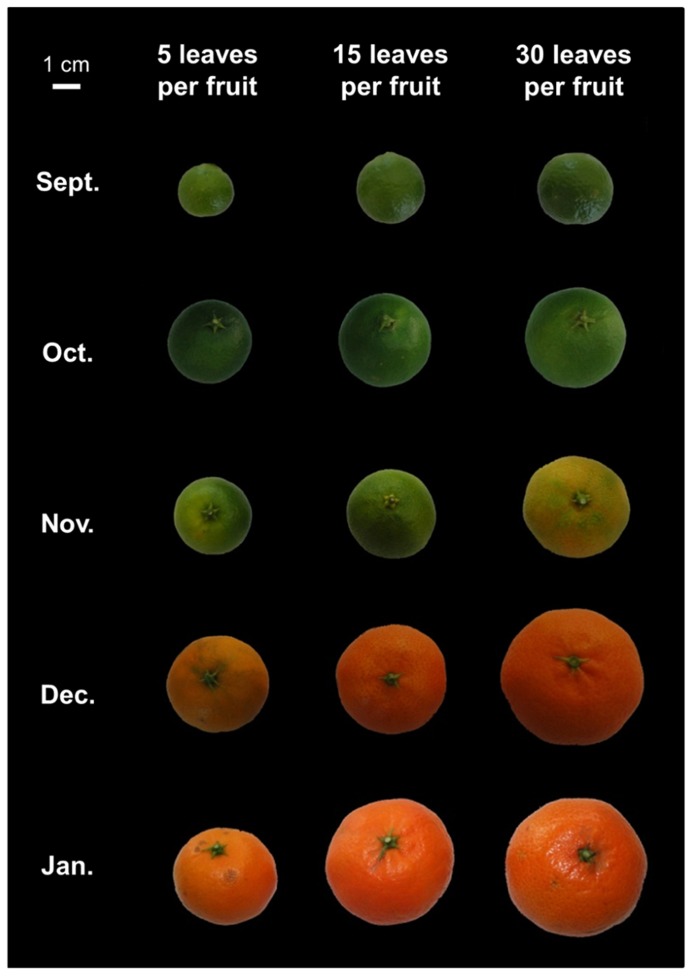
**Photographs of representative clementine fruits from fruiting branches bearing 5, 15, or 30 leaves.** The pictures illustrate the effects of carbon availability on fruit growth and maturity. A low carbon supply (five leaves per fruit) reduced fruit diameter and induced a delay in fruit ripening.

**FIGURE 6:**
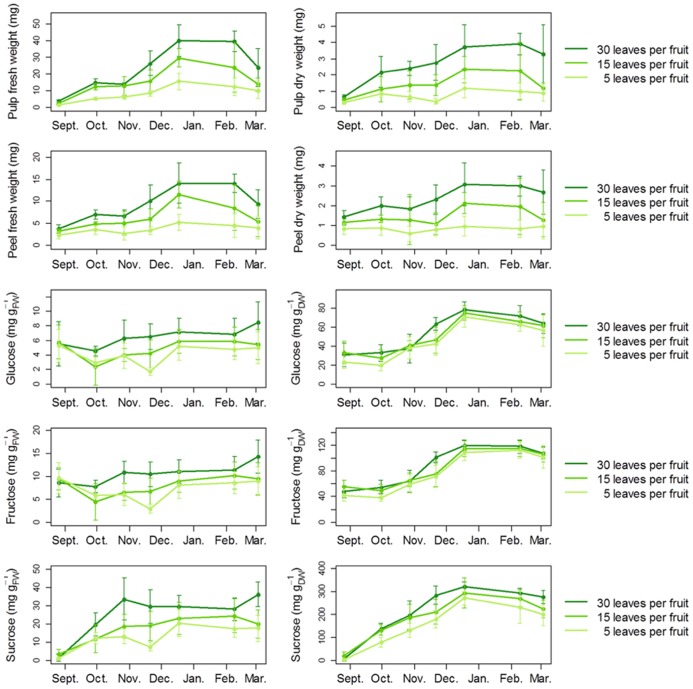
**Developmental changes in clementine fruit growth and soluble sugar contents according to carbon availability.** To probe the effect of carbon availability on fruit growth in field conditions, a defoliation treatment was applied on girdled fruiting branches from adult clementine trees to obtain three levels of leaf-to-fruit ratio: 5, 15, and 30 leaves per fruit. The leaf-to-fruit ratio 30 corresponds to conditions of non-limiting carbon availability, and was considered as the control (Poiroux-Gonord et al., 2013). For masses and carbohydrate measurements, five fruits per level of leaf-to-fruit ratio were collected regularly in the morning (10:00, local time) from September to March during the 2008–2009 season. Error bars are 95% confidence intervals.

Carbon availability accelerated the onset of fruit color change (**Figure 5**), consistent with the idea that sugar availability promotes fruit ripening (Fanciullino et al., 2013). In the three treatments, full expansion and maturity were reached in the middle of December, as indicated by the plateau of fresh and dry weight, as well as sugar content (**Figure 6**). At later stages, these variables showed a moderate tendency to decrease, indicating over-maturity. These patterns are consistent with data from previous studies on clementine fruits growing under Mediterranean climate (Cercós et al., 2006; Tadeo et al., 2008). Whereas the defoliation treatment (performed in July) did not affect the seasonal pattern of fruit growth, it strongly affected fruit weight at maturity. For the 5 leaves per fruit treatment, a decrease of about 60 and 50% was observed in the pulp and peel fresh mass, respectively, when compared to the control. A less severe treatment also affected fruit mass, since a reduction in fresh mass up to 40% for both the pulp and the peel was observed at 15 leaves per fruit, when compared to the control. The fruit dry mass followed similar trends. The defoliation treatment also modified soluble sugar contents in pulp (**Figure 6**). The changes in soluble sugars were mainly due to variations in sucrose concentrations. The largest decrease in sucrose was observed for the five leaves per fruit treatment in November, with a reduction of 80% when expressed on a fresh weight basis.

The strong, positive impact of the leaf-to-fruit ratio on fruit weight and sugar contents indicates that fruit growth is positively controlled by carbon availability, as repeatedly observed in other tree species (e.g., Berman and DeJong, 1996, on peach; Léchaudel et al., 2007, on mango). These results are consistent with the fruit as a sink organ relying on proximal leaves for its supply with photoassimilates.

#### Atmospheric water deficit highlights the osmotic role of photoassimilates on fruit growth

**FIGURE 7:**
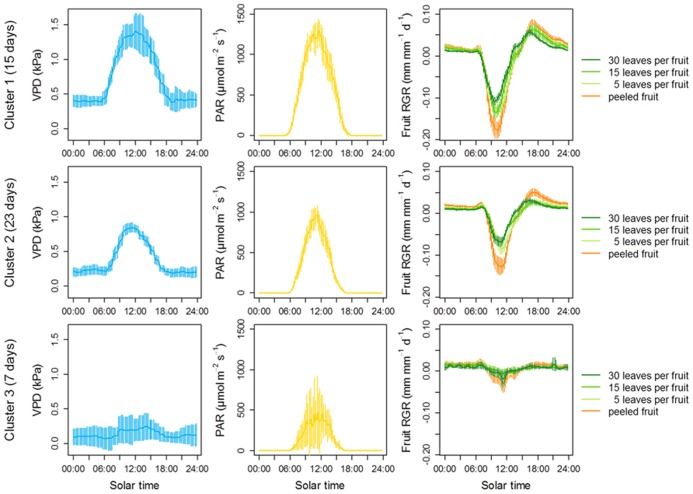
**The relationship between carbon availability and fruit growth is conditional on atmospheric water stress in clementine.** Fruit growth was monitored using displacement transducers on at least three fruits per level of leaf-to-fruit ratio during 45 days between early September and late November. Some displacement transducers were also placed on peeled fruits from girdled fruiting branches with 30 leaves. Climate data (air humidity, temperature and irradiance) were collected every 15 min together with changes in fruit diameter. A k-means clustering was performed to group days of the experiment on the basis of their similarity in VPD. Three clusters were statistically obtained, equivalent to days with a dry (top), intermediate (middle), and wet (bottom) atmosphere. Irradiance as well as fruit growth was then averaged according to these three clusters. Error bars are 95% confidence intervals.

To evaluate how moderate water deficit may affect the relationship between fruit growth and carbon availability, we took advantage of the daily, natural fluctuations in the VPD of the atmosphere. Daily growth variations were monitored in fruits at the three levels of carbon availability previously described. In addition, growth was monitored on fruits which were carefully peeled and grown at high carbon availability, in order to increase fruit exposure to evaporative demand. Our growth analysis covered 45 days between early September and late November. Throughout this period, daily patterns of VPD were highly variable from day to day, exhibiting a peak at midday during the driest days, and remaining almost constant during the wettest days. A k-means clustering analysis was performed to partition the days according to their similarity in VPD and three clusters were obtained gathering days with the highest (VPD_max_ > 1 kPa), intermediate (1 kPa < VPD_max_ < 0.5 kPa), and lowest VPD (VPD_max_ < 0.5 kPa), respectively. The fruit growth patterns were then averaged within each of these three clusters (**Figure 7**).

Consistent with the negative effect of VPD on fruit water potential, strong, negative depressions of fruit growth were observed at high VPD whatever the leaf-to-fruit ratio. Negative expansive growth is typically due to a water loss that leads to fruit shrinkage (Fishman and Génard, 1998). The strongest depressions of growth were observed in the peeled fruits even though they were associated with the high leaf-to-fruit ratio, suggesting a prevailing hydraulic origin of these depressions. Furthermore, these depressions were attenuated at moderate VPD and completely abolished at low VPD. After these brutal depressions, fruit diameter progressively recovered from ca. 11:00 in the morning, and diameter variation became positive again 3 h later. This rapid recovery was probably due to either a midday depression of stomatal conductance (Hu et al., 2009), a rapid osmotic adjustment permitted either by newly synthesized photoassimilates or by ion uptake from the cell wall (Thorpe et al., 1993), or a rapid softening of cell walls (Bogoslavsky and Neumann, 1998). Remarkably, the strongest depressions of fruit growth were observed during the periods of highest irradiance, which co-occurred with the periods of highest VPD (**Figure 7**). This result suggests that, at least on the short-term, the negative effect of evaporative demand on fruit growth dominates over the positive effect of irradiance.

Interestingly, a high leaf-to-fruit ratio reduced the maximum fruit shrinkage. Moreover, this effect was clearly visible at high VPD, but was strongly reduced at intermediate VPD and no longer visible at low VPD (**Figure 7**). An explanation for the positive effect of carbon availability on preventing fruit shrinkage under atmospheric water deficit is that higher concentrations of photoassimilates contribute to lower osmotic potential and reduce water loss. Such a hypothesis is supported by literature data. In peach fruit, a high leaf-to-fruit ratio increased the osmotic pressure of the fruit and reduced the transpiration-induced fruit shrinkage, either during experiments (McFadyen et al., 1996) or *in silico* using a biophysical model of fruit growth (Fishman and Génard, 1998). In line with this, although pulp sugar contents were relatively unaffected by the leaf-to-fruit ratio when expressed per unit of dry weight, they were clearly increased by a high leaf-to-fruit ratio when expressed per unit of fresh weight, i.e., when considered as solutes (**Figure 6**). These concentrations were high enough to postulate an osmotic effect. Thus, our results suggest that the positive effect of carbon availability on short-term fruit growth under air water deficit is not linked to a limitation of structural growth by carbon under these conditions but rather the result of a soluble carbohydrate-induced lower osmotic potential which buffers the variations in fruit diameter generated by the fluctuations in water potential.

## CONCLUSION

Increases in carbohydrate concentration following moderate water stress raise the question of the role of carbohydrate availability on plant growth under water deficit. In this study, we addressed this question using two different species under either soil or air water deficits. In growing *Arabidopsis* leaves, soil water deficit induces an accumulation of carbohydrates which contributes to shift growth during the nighttime, a period when water balance is much less affected by transpiration. In mutants impaired in starch metabolism, growth at night is even promoted by water deficit, suggesting that water stress induces carbon satiation in these growing leaves. In clementine fruits, atmospheric water stress-induced depressions of growth are of hydraulic nature and deepen during dry days despite higher irradiance. A high carbohydrate status reduces these depressions, suggesting that photoassimilates are rapidly mobilized and used as osmotica to buffer turgor pressure against the variations in fruit water balance. These two rather different systems illustrate that plants under moderate water stress do not suffer from carbon starvation which would impair structural growth, but instead make an efficient use of their carbohydrates to buffer expansive growth variations against the environmental fluctuations in water availability.

## Conflict of Interest Statement

The authors declare that the research was conducted in the absence of any commercial or financial relationships that could be construed as a potential conflict of interest.

## AUTHOR CONTRIBUTIONS

All authors designed and performed the experiments. Florent Pantin, Anne-Laure Fanciullino, Catherine Massonnet, and Bertrand Muller wrote the manuscript.
